# Around the Hospitals

**Published:** 1894-08-04

**Authors:** 


### Around the Hospitals.

[Notice to the Managers of Institutions.—Special space is now reserved for the insertion of notices of hospital meetings and festivals. The Editor requests that all notices may reach The Hospital Office, 428, Strand, at least one week before the date of each meeting.]

**The Royal Mineral Water Hospital, Bath.**—This institution has existed since 1742, for the treatment of those who are likely to benefit by a course of treatment at Bath, and who cannot afford to secure the benefits for themselves. The hospital now contains 171 beds. Many of the usual tiresome preliminaries to obtain admission into a hospital are absent here, the only condition being that the patient should be poor and suitable for treatment, and they are then admitted free of expense when a vacancy exists. Every year a larger number seek admission, for the fame of Bath waters is a matter of history, and does not diminish. The patients come from all parts of England. Upwards of 1,800 patients were treated last year. Annual subscriptions have shown a falling off, and the same is also observable, but to a lesser respect, in donations. The expenditure was £5,045.

**The Royal Bath Hospital, Harrogate.**—The Harrogate hospital has been always a useful institution, as its natural advantages enable it to afford much relief to sufferers from rheumatism and kindred diseases. Since the establishment of the Rawson Convalescent Home in connection with it more useful still has it become. Rheumatism may often be regarded as a disease of the convalescent, needing treatment no less than those forms of ailment which confine a patient to bed. Harrogate is now able to receive both classes of patients into whichever of the two establishments is most suitable for the case. Governors have the right of recommending, and their recommendations reduce the at any time moderate charge made for treatment. Patients can also be received without a recommendation, on payment of the full charges. The number of patients received last year in both institutions was 1,020. The record for the home, however, covered only eight months. The average cost per patient for a stay of three weeks is £2 2s. 7d. The hospital is in the fortunate position of closing the year with a credit balance.

**The Liverpool Royal Infirmary.**—Liverpool has in its infirmary an institution worthy of so important a centre. Like the city itself, it shows no signs of diminishing its activity, but is increasing in its work so rapidly that its financial resources are proving far too small. The Liverpool Infirmary, like many other charities in manufacturing districts, is finding that the conversion of industries into companies is depriving them of the aid of the wealthy employers, who were responsible and self-respecting individuals, contributing towards charitable schemes which were liable to assist their employés, in whom they took a personal interest. It may be that as much or more money is made in Liverpool now than formerly, but there is a greater absence of personal interest and even local expenditure. Owing to the activity of the managers of the infirmary, annual subscriptions have decidedly increased; but the increase is not yet sufficient to cope with the work. During the past year the in-patients numbered 3,310, and the out-patients 12,724. The income of the infirmary was £19,172, of which £2,876 was provided by a transfer from the maintenance fund, required to meet the expenditure.

